# Fear of COVID-19 is associated with trust, subjective numeracy, and differentially with loneliness in older versus younger adults

**DOI:** 10.3389/fpsyg.2023.1080631

**Published:** 2023-02-08

**Authors:** Alison F. Chung, Madeleine J. Teasell, Valentina Pergher, Allen E. Thornton, Wendy Loken Thornton

**Affiliations:** ^1^Department of Psychology, Simon Fraser University, Burnaby, BC, Canada; ^2^Department of Psychology, Harvard University, Cambridge, MA, United States

**Keywords:** COVID-19 fear, loneliness, interpersonal trust, subjective numeracy, age, gender

## Abstract

**Introduction:**

The emotional impacts of the COVID-19 pandemic and resulting public health emergency are only beginning to be understood.

**Methods:**

We assessed the contributions of emotional and cognitive factors and age-related comorbidities to greater COVID-19 fear in a community dwelling sample of 142 younger (M_age_ = 19.63, SD_age_ = 2.59) and 157 older (M_age_ = 72.01, SD_age_ = 7.06) adults, between July 2020 and July 2021. We hypothesized that individuals with increased loneliness, depression, and/or decreased subjective numeracy (SN) and interpersonal trust would experience more COVID-19 fear. We also predicted that females and older adults would experience more COVID-19 fear given that age-related comorbidities are associated with increased illness severity.

**Results:**

Results showed that the extent of loneliness in older adults was more strongly related to fear of COVID-19 than it was in younger adults (β = 0.197, *p* = 0.016), and poorer SN was associated with increased COVID-19 fear in both age groups (β = −0.138, *p* = 0.016). Further, higher interpersonal mistrust was associated with increased COVID-19 fear (β = 0.136, *p* = 0.039), as was identifying as female (β = 0.137, *p* = 0.013).

**Discussion:**

Given that self-described poor numeracy was a marker for greater COVID-19 fear, investigators and policy makers might consider mitigation opportunities addressing data literacy requirements imposed by the media. Further, outreach to mitigate loneliness, particularly of the elderly, might effectively lessen the negative psychological impact of this ongoing public health crisis.

## 1. Introduction

The coronavirus disease (COVID-19) pandemic triggered a host of unprecedented changes to societal function (WHO, 2022). In response to outbreaks, emergency lockdown orders, social distancing measures, and orders to quarantine were invoked (e.g., WHO^1^). The isolating nature of these orders led to negative impacts on the psychological well-being of individuals of all ages (Best et al., 2021; Gill et al., 2021). With the contagious yet invisible nature of COVID-19, a psychosocial toll is placed on individuals regardless of whether they have contracted the illness (Ahorsu et al., 2020).

During the initial pandemic response, reports of elevated distress related to COVID-19 were evident (Best et al., 2021; Varga et al., 2021; Lee et al., 2022). Extant literature on fear of COVID-19 has yielded contrasting findings, with some suggesting greater fear of COVID-19 in younger adults (Bäuerle et al., 2020; Quadros et al., 2021) and others reporting no age differences (Luo et al., 2021). Preliminary research reports positive associations between fear of COVID-19 and greater depressive and anxiety symptoms (Fitzpatrick et al., 2020). Fear of COVID-19 may also be related to loneliness (Enea et al., 2021) although its potential impact across age groups is not well understood.

Recent findings reported greater loneliness in younger adults compared to older adults during the pandemic (Bu et al., 2020; Wickens et al., 2021; McDonald et al., 2022), which contrasts established pre-pandemic findings of greater loneliness in older adults (Gerst-Emerson and Jayawardhana, 2015). Early in the pandemic, younger adults reported higher rates of depression, anxiety, loneliness, and post-traumatic stress (Lee et al., 2020; Statistics Canada, 2021) and reported lower perceived coping efficacy compared to older adults (Carstensen et al., 2020; Klaiber et al., 2021). This latter age difference was attributed to increased emotional well-being in older adults, as well as to increased daily stresses (i.e., work, family) for younger adults (Carstensen et al., 2020; Klaiber et al., 2021). Nonetheless, in the last 2 years, older adults reported greater loneliness and depressive symptoms compared to that reported before the pandemic (Kotwal et al., 2020; Raina et al., 2021; Alhalaseh et al., 2022). Furthermore, those who experienced increased feelings of loneliness were more likely to report greater depression, anxiety, and fear of worsening health (Kotwal et al., 2020). As social isolation in older adults is a concern that existed prior to the pandemic, it is worrisome to find that loneliness has increased during the pandemic given the known negative impacts including reduced psychological well-being, poorer physical health, and higher mortality (Leigh-Hunt et al., 2017).

Additionally, physical health status may modify the risk of becoming severely ill from COVID-19, and thus may increase COVID-19 fear (USA: Centers for Disease Control and Prevention, 2022). Diabetes, for example, is reportedly the most impactful comorbidity in predicting COVID-19 disease severity, while hypertension (HTN) and high cholesterol exacerbate disease severity and increase fatality (Rod et al., 2020; Wolff et al., 2021; Centers for Disease Control and Prevention, 2022). Furthermore, patients with high-risk diseases such as diabetes, HTN, and cardiovascular disease, demonstrated increased COVID-19-related fear compared to healthy individuals (Kohler et al., 2021; Musche et al., 2021). Age-related vulnerability is exemplified by the older adult contribution to national COVID-19 deaths. During 24-months of the pandemic (2020 and 2021), 93% of COVID-19 related deaths were in older adults. While COVID-19 related deaths were higher in males compared to females (57% male, 43% female) (WHO, 2022), findings report that females report greater COVID-19 fear (Alsharawy et al., 2021).

Sociocultural factors may also be related to fear of COVID-19. With the introduction of stay-at-home orders, media and internet consumption grew exponentially. Indeed, the Canadian Internet Use Survey revealed that 90% of those aged 15–35 years and 35% of those aged 65–75 years increased online activities during late 2020 to early 2021 (Bilodeau et al., 2021). Further, the frequency, duration and diversity of COVID-19 media exposure was associated with greater anxiety symptoms (Wheaton et al., 2021). Importantly, the ubiquitous COVID-19 news was steeped in rapidly evolving, dense, and often ambiguous statistics. Several studies have reported that comprehension of health-related statistical concepts tends to be poor among the public (see Rolinson et al., 2020). Subjective numeracy (SN; subjective perception of one’s arithmetic capacity) is strongly associated with statistical numeracy, which is required for making informed decisions and understanding health risks (Rolison et al., 2013; Rolinson et al., 2020). Past work has shown that in those with poorer SN, susceptibility to framing effects (e.g., perceiving a 15% mortality rate as worse than an 85% vitality rate despite both presenting the same information) is associated with increased anxiety and fear (Levin et al., 1998; Rolinson et al., 2020). Moreover, it has been shown that lower SN is associated with lower scores on a measure of COVID-19 knowledge (Zamarian et al., 2021), supporting the ideas that reduced SN could be associated with greater fear of COVID-19.

One last factor that has been suggested to play a fundamental role in adherence to health directives and in susceptibility to misinformation is trust. A high level of trust in the government and society is associated with higher vaccination rates as observed in a sample of 177 countries with vaccine availability (COVID-19 National Preparedness Collaborators, 2022). In a study of 127 countries, greater trust in the government and science was associated with decreased mortality, although greater trust in society was associated with increased mortality (Reiersen et al., 2022). The increased mortality was felt to potentially reflect the misplaced trust of others to stay home/report symptoms when sick. Not all members of society are truthful, and this might impact mortality (Reiersen et al., 2022). Nonetheless, while the emergent findings on institutional trust appear consistent within the context of COVID-19, there has been little work on the role of interpersonal trust (see Wollebæk et al., 2021), with some studies showing both negative (Iacono et al., 2021) and positive (Kye and Hwang, 2020) effects of the pandemic on interpersonal trust. Regarding fear of COVID-19; those with decreased interpersonal trust may be expected to show increased fear, as those who experience greater worry may be less trusting of groups (Jørgensen et al., 2021), and tend to be more adherent to social distancing guidelines (Oosterhoff and Palmer, 2020). To date, we are aware of no reports that have employed relevant multivariate models to examined trust in predicting COVID-19 fear. Thus, the relative importance of interpersonal trust remains obscure.

The current study was designed to clarify several issues pertinent to fear of COVID-19. First, we hypothesize that regardless of age, increased loneliness and depression will be associated with increased fear of COVID-19. Likewise, we predict that participants with lower versus higher SN would show greater COVID-19 fear. Further, we investigate the contribution of interpersonal trust to COVID-19 fear, anticipating age invariance for this effect. Finally, we expect that fear of COVID-19 is higher in females, as has been reported for anxiety symptoms.

Age was targeted specifically for its contribution to COVID-19 fear to address this apparent gap in the literature. We hypothesized that older adults would experience greater COVID-19 fear given their known vulnerabilities. We also explore whether age group moderates the anticipated effects of loneliness, depression, SN, and trust. Finally, the presence of diseases common in older adults might portend greater fear of COVID-19. Consequently, we explored whether older adults treated for diabetes (Wild et al., 2004), HTN (Hajjar and Kotchen, 2003), and high cholesterol (Wong et al., 2006) suffer from greater COVID-19 fear than older adults not undergoing treatment.

## 2. Materials and methods

### 2.1. Participants

#### 2.1.1. Exclusion’s criteria

This study was conducted between July 2020–July 2021 during waves 2 and 3 of the COVID-19 pandemic. Participants were considered eligible for inclusion if they met the minimum age requirement for each age group (17 years for younger adults; 60 for older adults). Participants who did not meet the English language eligibility requirement, assessed through four English as a Second Language questions, were excluded from the study. Participants who did not complete the fear of COVID-19 Scale and who had missing values were also excluded from the study, resulting in 305 total participants, 5 of whom were excluded for missing data, and 1 was excluded as they emerged as an extreme outlier for a total of *n* = 299.

#### 2.1.2. Sample characteristics

142 healthy younger adults (M_age_ = 19.63, SD_age_ = 2.59, 76.0% female) enrolled in introductory psychology courses were recruited through the Simon Fraser University Research Participation System. 157 healthy community-dwelling older adults (M_age_ = 72.01, SD_age_ = 7.06, 75.3% female) were recruited through snowball sampling, newspaper advertisement, and online advertisement (Facebook, Instagram, Craigslist.org). The rationale behind the division of the age groups has been guided by Goodman et al. (2021) and Zhang et al. (2022), that showed a significant difference in COVID-19 hospital admissions between young adults (10–29 years old; ≃ 2%) and older adults (60–79 years old; ≃ 20%) and a significant difference in COVID-19 death between young adults (10–29 years old; ≃ 1%) and older adults (60–79 years old; ≃ 22%). Furthermore, COVID-19 has been shown to disproportionally affect older individuals compared to young adults for its clinical manifestations, risk factors, and complications.

Older participants completed a 5-minute phone interview with a trained research assistant to verify eligibility and to provide additional information regarding their involvement. Eligible participants were sent a link to the Qualtrics questionnaire to complete remotely. Younger participants received course credits and older participants received a $20 Starbucks gift card or had $20 donated to the Canadian Red Cross’ Canadian Emergencies and COVID-19 Response Fund on their behalf. The study protocol was approved by the Simon Fraser University Research Ethics Board (#20200379), and all participants signed informed consent.

### 2.2. Measures

Participants completed a 1–1.5-hour remote testing session through Qualtrics Survey Software. The following measures were self-administered and were scored according to standardized procedures by trained research assistants.

#### 2.2.1. Demographics and health factors questionnaire

The Demographics portion of the self-report questionnaire asked participants about their demographic information (i.e., age, sex, ethnicity/race, language preferences). The Health Questionnaire, developed by the researchers (e.g., Yeung and Thornton, 2017; Walzak and Thornton, 2018) asked participants about the presence, absence, and treatment of diabetes mellitus (DM), HTN and high cholesterol/hypercholesterolemia (HC). Gender was self-identified as male, female, or other; no participants identified as other.

#### 2.2.2. Subjective numeracy (SN)

The Subjective Numeracy Scale (SN) is an 8-item self-rated measure of participants’ understanding of basic mathematics and probability (Fagerlin et al., 2007). Participants indicated their subjective perception of arithmetic proficiency and preference for numbers of words on a 5-point scale. Questions do not include mathematical problems or equations. Total scores range from 8 to 48, with higher scores representing greater SN ability. The SN scale has been validated using a sample stratified to mirror the United States population based on age, gender, race, education level, and income (Zikmund-Fisher et al., 2007).

#### 2.2.3. Loneliness (UCLA-L)

The University of California, Los Angeles (UCLA) Loneliness Scale Version 3 (Russell, 1996) is a 20-item self-rated measure of subjective feelings of loneliness and isolation. Participants indicated how frequently each statement describes them on a 4-point Likert scale ranging from “Never” to “Often.” Total scores range from 20 to 80, with higher scores representing greater feelings of isolation and loneliness. The UCLA Loneliness scale has demonstrated high internal consistency and reliability across age groups (Russell, 1996).

#### 2.2.4. Depression (CES-D)

The Center for Epidemiological Studies Depression Scale (CES-D; Radloff, 1977) is a 20-item self-rated measure of depressive symptoms. Participants indicated how they have felt or behaved during the past week on a 4-point Likert scale ranging from “Rarely or none of the time” to “Most or all the time.” Total scores range from 0 to 60, with higher scores representing more severe depressive symptomatology. The CES-D scale has demonstrated high internal consistency and adequate reliability in adults (Cosco et al., 2017).

#### 2.2.5. Fear of COVID-19

The fear of COVID-19 Scale (Ahorsu et al., 2020) is a 7-item self-rated measure of COVID-19 fear. Participants indicated their agreement with presented statements on a 5-point Likert scale ranging from “Strongly disagree” to “Strongly agree.” Total scores range from 7 to 35, with higher scores representing greater COVID-19 fear. The fear of COVID-19 scale has demonstrated acceptable initial validity and reliability in adults (Ahorsu et al., 2020).

#### 2.2.6. Trust scale

The Trust Scale (Inglehart et al., 2014) is a 7-item self-rated measure of trust and subjective trustworthiness for six different groups including family, friends, neighbors, strangers, and people of different religions and nationalities. Participants indicated their agreement with presented statements on a 4-point Likert scale ranging from “Trust completely” to “Do not trust at all.” Total scores ranged from 6 to 24, with lower scores representing higher trust.

### 2.3. Statistical analyses

All analyses were conducted using IBM SPSS Statistics software (v. 27^2^). Hierarchical linear regression was used to determine the contribution of the primary continuous variables of interest to fear of COVID-19 (i.e., CES-D, SN, UCLA-L, and Trust). As well, Gender, Age Group, and the Age Group moderating effects on continuous variables were evaluated. Age group and Gender were entered on the first Block. On the second Block centered scores for CES-D, SN, UCLA-L, and Trust were entered. On the final block the interaction terms were entered. The final model involved refinement by deletion of all non-contributory Block 2 terms (*p* > 0.10) and any non-significant Age Group interactions (*p* > 0.05).

Independent sample *t*-tests were used to explore mean differences in the fear of COVID-19 experienced by older adults with and without DM, HTN, and HC. As expected, we did not explore these effects in younger adults as there were too few younger adults with these conditions.

## 3. Results

### 3.1. Descriptive statistics and correlations

Table 1 presents participant characteristics for each age group. Means and standard deviations are reported for age, education, Fear of COVID-19, CES-D, SN, UCLA-L, and Trust scores. Frequencies and percentages are reported for gender and ethnicity.

**TABLE 1:** Characteristics and demographics of the young and older adults, and full sample.

	Younger adults *N* = 142	Older adults *N* = 157	Full sample *N* = 299
Age (Mean ± SD) Median (IQR)	19.63 ± 2.59 19.0 (2.00)	72.01 ± 7.06 72.0 (10.00)	47.13 ± 26.74 61.0 (53.0)
Education (Mean ± SD) Median (IQR)	13.42 ± 1.52 13.0 (2.00)	16.34 ± 2.94 17.0 (5.00)	14.96 ± 2.79 15.0 (5.00)
Ethnicity % (Cauc/Asian/SE* Indian/Other)	25.9/34.5/30.2/9.9	87.7/5.8/1.9/4.6	58.5/19.4/15.3/6.8
Female %	108 (76.1%)	119 (75.8%)	227 (75.9%)
Fear of COVID-19 Median (IQR)	15.94 ± 5.59 16.0 (9.25)	15.01 ± 5.21 15.0 (8.50)	15.45 ± 5.40 15.00 (8.00)
CESD* (Mean ± SD) Median (IQR)	22.48 ± 11.35 22.0 (17.25)	9.38 ± 8.14 7.5 (10.00)	15.66 ± 11.78 12.5 (17.00)
UCLA* (Mean ± SD) Median (IQR)	46.41 ± 11.48 46.0 (17.25)	36.38 ± 11.37 34.0 (15.00)	41.14 ± 12.45 39.0 (18.00)
SN (Mean ± SD) Median (IQR)	31.06 ± 7.74 32.0 (10.00)	34.04 ± 9.08 35.0 (13.50)	32.62 ± 8.59 34.0 (12.00)
Overall trust of others (Mean ± SD) Median (IQR)	14.54 ± 3.05 15.0 (5.00)	11.35 ± 2.10 11.0 (3.00)	12.86 ± 3.04 13.0 (4.00)
Presence of DM* (N / %)	–	10 (6.40%)	–
Presence of HTN* (N / %)	–	45 (28.7%)	–
Presence of HC* (N / %)	–	39 (24.8%)	–

*SE, South East; CESD, Center for Epidemiological Studies Depression Scale; UCLA, University of California, Los Angeles Loneliness Scale; DM, diabetes mellitus; HTN, hypertension; HC, high cholesterol/hypercholesterolemia; SD, standard deviation.

### 3.2. Main analysis

#### 3.2.1. Preliminary regression model

Hierarchical regression revealed that depressive symptoms as measured by the CES-D was non-contributory, i.e., it provided no main or interactive effects in accounting for variance in the fear of COVID-19. All other Block 2 variables (UCLA-L, SN, Trust) were retained. In Block 3, we tested age moderation of the other independent variable effects (i.e., interactions. Age group did not interact with SN or Trust in contributing to fear of COVID-19.

#### 3.2.2. Final regression model

**FIGURE 1:**
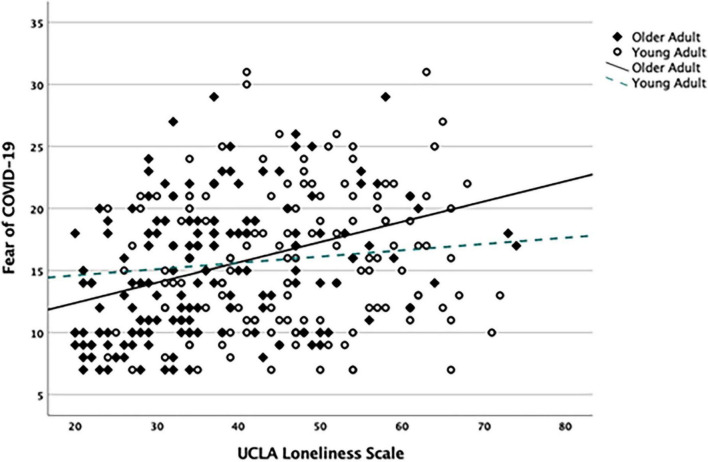
Moderating effects of age group on the relationship between fear of COVID-19 and loneliness.

Table 2 presents the results of the final hierarchical regression analysis (*N* = 299). This model includes the only significant age interaction (i.e., Age group by loneliness). A main effect of SN on fear of COVID-19 (β = −0.138, *p* = 0.016) was revealed, indicating that lower SN was associated with higher fear. In addition, Age group interacted with loneliness (UCLA-L) (β = 0.197, *p* = 0.016) (Figure 1). The results showed that greater loneliness has a larger effect on the fear of COVID-19 in older relative to younger adults, indicating the moderating effects of age group on loneliness. Further, lower interpersonal trust was associated with an increased COVID-19 fear (β = 0.136, *p* = 0.039), as was identifying as female (β = 0.137, *p* = 0.013).

**TABLE 2:** Final hierarchical regression of age group, loneliness, subjective numeracy, and trust scores on fear of COVID-19.

Predictor	B*	S.E.*	β*	*t*	*p*	R^2^*	ΔR^2^*
Block 1						0.032	0.032
Age group	-0.926	0.618	–0.086	-1.498	0.135		
Gender	1.986	0.722	0.157	2.751	0.006		
Block 2						0.118	0.086
Age group	1.035	0.724	0.096	1.430	0.154		
Gender	1.670	0.701	0.132	2.381	0.018		
Loneliness	0.093	0.027	0.214	3.503	0.001		
Numeracy	-0.084	0.036	–0.133	-2.336	0.020		
Trust	0.244	0.118	0.137	2.065	0.040		
Block 3						0.136	0.017
Age group	0.960	0.719	0.089	1.335	0.183		
Gender	1.732	0.696	0.137	2.488	0.013		
Loneliness	0.028	0.038	0.064	0.739	0.460		
Numeracy	-0.087	0.036	–0.138	-2.427	0.016		
Trust	0.243	0.117	0.136	2.071	0.039		
Age group × Loneliness	0.125	0.052	0.197	2.415	0.016		

*B, regression slope; S.E., standard error; β, beta coefficient; R^2^, coefficient of determination; ΔR^2^, R^2^ adjusted.

### 3.3. Exploratory analysis

Independent sample *t*-tests were conducted in older adults to determine the association between health status variables with fear of COVID-19. A small group of older adults (*n* = 10) who reported being currently treated for DM showed a trend to report higher fear of COVID-19 (*p* < 0.066; Hedge’s *g* = −0.53) compared to those who did not report treatment for DM (*n* = 147). There were no differences in fear of COVID-19 scores for either HTN or HC (*p*s > 0.50; g < 0.01).

## 4. Discussion

Overall, this study conducted during waves 2 and 3 (2020–2021) of the COVID-19 pandemic, showed that older adults experienced greater COVID-19 fear related to loneliness as compared to younger adults. Furthermore, we found that both younger and older individuals with a greater COVID-19 fear reported lower SN scores, and a link between interpersonal trust and COVID-19 fear. Specifically, increased interpersonal trust was associated with less COVID-19 fear. Finally, our outcomes shed light on the impact of other diseases during the COVID-19 pandemic. In this case, older adults treated for DM experienced a greater fear of COVID-19 compared to those without DM, while the presence of other diseases, such as HTN and HC, did were not associated with fear of COVID-19. Below, we discuss each of these findings in turn.

### 4.1. Primary analysis discussion

#### 4.1.1. Loneliness and fear of COVID-19 in adults

Despite finding no significant mean differences in COVID-19 fear in older versus younger participants, older adults experienced COVID-19 fear as a stronger function of loneliness compared to that of younger adults. Given that in older versus younger adults COVID-19 fear appears more contingent upon loneliness, interventions might be focused accordingly. This aligns with our hypothesis suggesting that older adults are especially vulnerable to psychosocial consequences of stay-at-home orders and isolation caused by the COVID-19 pandemic, which may lead to decreased quality of life and mental well-being (Rantakokko et al., 2010; Rantanen, 2013), as well as declines in cognitive functioning (Braley et al., 2022).

#### 4.1.2. Subjective numeracy and fear of COVID-19 in adults

The current findings also present novel associations between SN and fear of COVID-19 in both older and younger adults. Specifically, we revealed that those reporting lower SN experienced greater fear of COVID-19. While prior studies have reported that lower subjective numeracy is associated with lower scores on a measure of COVID-19 knowledge (Zamarian et al., 2021), and to lesser ability and confidence in interpreting statistical information regarding general health risks (Rolinson et al., 2020), the current findings extend these associations to older adults and to increased fear of COVID-19 during this pandemic. These findings suggest that investigators and policy makers might consider mitigation opportunities addressing data literacy requirements that accommodate those experiencing such concerns and/or limitations.

#### 4.1.3. Trust, gender and fear of COVID-19 in adults

Past research on the role of trust and COVID-19 behaviors is often both contradictory and dependent on the measures used (see Wollebæk et al., 2021). However, previous research has demonstrated that variations in societal and interpersonal trust influenced one’s compliance to health directives and behaviors during the pandemic. Interestingly, increased interpersonal trust has been associated with greater COVID-19 related mortality, which was interpreted as reflecting lower fear of the illness in those who were more trusting in other’s behaviors (Reiersen et al., 2022). Our observation confirmed that greater interpersonal trust is associated with less COVID-19 fear, providing support that individuals with more trust in others experience less fear of COVID-19.

Lastly, as we predicted, despite that fact that COVID-19 related deaths have been reported to be higher in males compared to females (WHO, 2022), our findings are consistent with a previous report (Alsharawy et al., 2021) showing that females report greater fear of COVID-19. This finding is consistent with numerous reports of higher rates of anxiety symptoms and disorders in women across age groups (see Remes et al., 2016 for a review).

### 4.2. Secondary analysis discussion

#### 4.2.1. Diabetes mellitus (DM) and fear of COVID-19 in older adults

In the older adults, being treated for DM was associated with greater fear of COVID-19, given the medium sized effect, compared to those without DM. The presence of HTN and HC were associated with trivial effects (*g*’s < 0.11). While the DM effect (*g* = 0.553, *p* = 0.066) did not reach traditional statistical significance, the magnitude suggests that in those few older participants with DM (*n* = 10), their fear may be considered meaningfully higher in this exploratory analysis while other select health conditions were not contributory. This is unsurprising given that DM has been associated with nearly a two-fold increase in both morbidity and severity of COVID-19 compared to those without DM (e.g., Kumar et al., 2020).

## 5. Conclusion and future directions

The public has been presented with a multitude of health-related decisions such as choosing to social distance, wear a mask, or get vaccinated. Misinterpreting data and statistics presented in the media or choosing to not follow government directives due to mistrust may influence one’s susceptibility to and ultimately fear of COVID-19. Despite the need for health directives to keep society safe, measures should be taken to mitigate these consequences, especially with the most vulnerable populations (Gerst-Emerson and Jayawardhana, 2015; Statistics Canada, 2021). At appropriate levels, healthy fear of COVID-19 may be a positive pandemic adaptation by decreasing risky health behaviors, but at heightened levels it may entail suffering. When experiencing increased fear of COVID-19, reactions may arise such as a phobia of being infected, stress, and further weakening of the immune system due to the increased psychological toll (Arora et al., 2020). Further, current findings suggest that education programs aiming to improve individual’s emotions and self-evaluation of abilities (Rolinson et al., 2020) as well as public awareness campaigns providing the public with more confidence in the accuracy of news may be beneficial. Such interventions could include exposing false news as soon as it is discovered and ensuring that people in positions of power demonstrate the abidance to health advice the government is promoting would play a fundamental role (Ng and Kemp, 2020).

The current findings should be understood within the context of certain limitations. Perhaps most apparent is that directionality is assumed, as it is indeterminant in cross-sectional datasets. For instance, it might well be that fear of COVID-19 leads to greater loneliness and/or to greater anxiety that erodes confidence and SN. Bidirectionality is also possible. Indeed, while the association of SN and fear of COVID-19 is novel and merits further study, SN is subjective by definition and influenced by the participant’s confidence and self-perception. Thus, SN may also have captured other subjective aspects of the individual apart from numeracy, such as self-efficacy or general confidence. Further, a limitation regarding health risk factors is that we had very few persons with DM in this study (*n* = 10), which, despite the moderate effect size noted, renders the reliability of this finding questionable until replication. While our outcome measure of fear of COVID-19 was specifically designed to assess fear related to the ongoing pandemic (Ahorsu et al., 2020), we cannot conclude from the current study if these associations are limited to issues specific to COVID-19 or if they generalize to other health-related or general anxieties. Finally, further studies on this topic with larger and more representative samples should investigate the potential moderating effects of loneliness with age-related comorbidities.

## Data availability statement

The raw data supporting the conclusions of this article will be made available by the authors, without undue reservation.

## Ethics statement

The studies involving human participants were reviewed and approved by Simon Fraser University Research Ethics Board (#20200379). The patients/participants provided their written informed consent to participate in this study.

## Author contributions

AC wrote the initial draft and performed initial data screening and cleaning. MT performed the analyses. VP assisted with writing and administrative tasks. AT performed the analyses and assisted with writing. WT conceived the original idea and finalized the writing. All authors discussed the results and contributed to the final draft.

## Footnotes

1
https://www.who.int/emergencies/diseases/novel-coronavirus-2019

2
www.ibm.com
